# Anisotropy in ultrasound shear wave elastography: An add-on to muscles characterization

**DOI:** 10.3389/fphys.2022.1000612

**Published:** 2022-09-28

**Authors:** Ha-Hien-Phuong Ngo, Thomas Poulard, Javier Brum, Jean- Luc Gennisson

**Affiliations:** ^1^ Université Paris-Saclay, CEA, CNRS, Inserm, BioMaps, Laboratoire d’Imagerie Médicale Multimodale à Paris-Saclay, Orsay, France; ^2^ Laboratorio de Acústica Ultrasonora, Instituto de Física, Facultad de Ciencias, Universidad de la República, Montevideo, Uruguay

**Keywords:** shear wave elastography, ultrafast ultrasound imaging, biomechanics, anisotropy, muscle

## Abstract

Ultrasound shear wave elastography was developed the past decade, bringing new stiffness biomarker in clinical practice. This biomarker reveals to be of primarily importance for the diagnosis of breast cancer or liver fibrosis. In muscle this biomarker become much more complex due to the nature of the muscle itself: an anisotropic medium. In this manuscript we depict the underlying theory of propagating waves in such anisotropic medium. Then we present the available methods that can consider and quantify this parameter. Advantages and drawbacks are discussed to open the way to imagine new methods that can free this biomarker in a daily clinical practice.

## 1 Introduction

At the end of the 20th century, static elastography was developed to enhance biological tissues characterization and medical diagnosis for pathological tissues (Ophir et al., 1991). It was inspired from the first clinical gesture of the physician, the manual palpation. It relied on the fact that the stiffness felt by the physician would translate the malignant nature of the tissue. Strain elastography was based on Hooke’s law, that links Young’s modulus (i.e. the stiffness *E*) to the applied stress (*σ*) and the observed strain (*ε*) in an isotropic and purely elastic media: *E* = *σ*/*ε*. Some studies have been performed with this technique on muscle (Akagi et al., 2011; Chino et al., 2012; Chino et al., 2014) However, the applied stress was difficult to quantify and, in practice, only strain profile was known leading to qualitative information. To overcome this limitation, the so-called shear wave elastography (SWE) based on shear wave propagation was developed. This approach assumes that, for a quasi-incompressible elastic solid, the shear wave speed (VS) is related to the shear modulus (µ) and to the Young’s modulus by the following relationship: *E* = 3*µ* = 3*ρV*_*S*_^*2*^, where ρ is the density of biological tissues (∼1,100 kg/m^3^). Numerous ultrasound-based shear wave elastography techniques were developed based on this physical hypothesis. These techniques involved two main basic steps: i) generating shear waves within a tissue and ii) imaging their propagation to retrieve their speed (Ophir et al., 1991a; Sarvazyan et al., 1995; Nightingale et al., 2002; Gennisson et al., 2003; Sandrin et al., 2003; Palmeri et al., 2008; McAleavey et al., 2009). With these “dynamic” techniques muscle has been widely investigated (Nordez et al., 2008; Akagi and Takahashi, 2013; Alfuraih et al., 2018; Hirata et al., 2020). Nonetheless, in order to solve the inverse problem (i.e. image tissue’s elasticity from shear wave velocity estimation), all these methods assume, in first approximation, that tissues are isotropic, quasi-incompressible and purely elastic media. However, this is not the case for muscles which can be modeled as transverse isotropic solid since they are composed of a random distribution of parallel fibers around one axis of symmetry (Eby et al., 2013). As a result, shear waves propagate faster along the muscle fibers than across the fibers (Gennisson et al., 2003; Miyamoto et al., 2015; Alfuraih et al., 2017). Thus, the link between Young’s modulus and shear waves speed is no more valid and the stiffness must be quantified by the estimation of two shear moduli, *µ*_*//*_ and *µ*_*⊥*_, along and perpendicularly to the fibers respectively (Royer et al., 2011). In order to improve tissue characterization and potentially medical diagnosis, it is then important to define new biomarkers such as anisotropy. As an example, because shear wave speed is linearly related to muscle force (Hug et al., 2015), estimating muscle anisotropic behavior would be of particular interest to better characterize muscle behavior and adaptation to various phenomena, such as training or disuse. In neuromuscular diseases, such as Duchenne myopathy, changes in the muscle molecular organization (Zemła et al., 2021) could certainly lead to modification of mechanical properties that could impact anisotropy. In this review, we focus on the possible ways to quantify anisotropy after considering theoretical aspects of shear wave propagation in a transverse isotropic medium. Then, an overview of the main dynamic ultrasound-based SWE techniques that have been developed from one-dimensional (1D) (one value) to three-dimensional (3D) (stiffness volume) shear wave imaging is presented and discussed with special emphasis on the developments made to include anisotropy as a new biomarker for physicians.

## 2 Shear wave propagation in transverse isotropic medium

Most muscles often present a random distribution of fibers oriented in the same direction, which implies a symmetry about the fiber orientation. Because of this symmetry, muscles are usually modeled as a transverse isotropic (TI) tissue, i.e. a tissue whose physical properties (e.g. stiffness) are symmetric with respect to an axis that is normal to the plane of isotropy.

In the limit of small displacements, the stress-strain relationship in an anisotropic tissue is linear and can be described by Hooke’s law

$$\sigma_{ij}{=C}_{ijkl}\varepsilon_{kl}$$ (1)

where *σ*_*ij*_ is the stress tensor, *ε*_*kl*_ is the infinitesimal strain tensor, *C*_*ijkl*_ is the fourth-order stiffness tensor. In Eq. 1 summation over repeated indices is implied (i.e. Einstein’s summation notation). In a TI tissue, based on symmetry, the eighty-one entries of the stiffness tensor are reduced to only five and Eq. 1 can be expressed using Voigt’s notation as follows:

$$\left[\begin{array}{c} \sigma_{1}\\ \sigma_{2}\\ \sigma_{3}\\ \sigma_{4}\\ \sigma_{5}\\ \sigma_{6} \end{array}\right]=\left[\begin{array}{cccccc} C_{11} & C_{12} & C_{13} & 0 & 0 & 0\\ C_{12} & C_{11} & C_{13} & 0 & 0 & 0\\ C_{13} & C_{13} & C_{33} & 0 & 0 & 0\\ 0 & 0 & 0 & C_{44} & 0 & 0\\ 0 & 0 & 0 & 0 & C_{44} & 0\\ 0 & 0 & 0 & 0 & 0 & C_{66} \end{array}\right]\left[\begin{array}{c} \varepsilon_{1}\\ \varepsilon_{2}\\ \varepsilon_{3}\\ \varepsilon_{4}\\ \varepsilon_{5}\\ \varepsilon_{6} \end{array}\right]$$ (2)

with $C_{66}=\left(C_{11}-C_{12}\right)/2$ . It is important to mention that in Eq. 2 the symmetry axis of the material (i.e. given by the direction of muscle fibers) is oriented parallel to ${\hat{x}}_{3}$ . Eq. 2 can also be expressed in terms of the Young’s moduli ( $E_{L}$ , $E_{T}$ ), Poisson’s ratios ( $\nu_{LT}$ , $\nu_{TT}$ ) and shear moduli ( $\mu_{L}$ , $\mu_{T}$ ), as

$$\left[\begin{array}{c} \varepsilon_{1}\\ \varepsilon_{2}\\ \varepsilon_{3}\\ \varepsilon_{4}\\ \varepsilon_{5}\\ \varepsilon_{6} \end{array}\right]=\left[\begin{array}{cccccc} 1/E_{T} & -\nu_{TT}/E_{T} & -\nu_{LT}/E_{L} & 0 & 0 & 0\\ -\nu_{TT}/E_{T} & 1/E_{T} & -\nu_{LT}/E_{L} & 0 & 0 & 0\\ -\nu_{LT}/E_{L} & -\nu_{LT}/E_{L} & 1/E_{L} & 0 & 0 & 0\\ 0 & 0 & 0 & 1/\mu_{L} & 0 & 0\\ 0 & 0 & 0 & 0 & 1/\mu_{L} & 0\\ 0 & 0 & 0 & 0 & 0 & 1/\mu_{T} \end{array}\right]\left[\begin{array}{c} \sigma_{1}\\ \sigma_{2}\\ \sigma_{3}\\ \sigma_{4}\\ \sigma_{5}\\ \sigma_{6} \end{array}\right]$$ (3)

where $\mu_{T}=\frac{1}{2}E_{T}/\left(1+\nu_{TT}\right)$ and the subscripts L and T correspond to the longitudinal and transverse directions relative to the to the symmetry axis, respectively.

A smart way to non-invasively measure the mechanical properties of TI tissue is to use wave propagation, since as in isotropic tissues, the wave speed is directly linked to the tissue’s stiffness constants or equivalently to the tissue’s Young and shear moduli. The fundamental difference between isotropic and TI tissue is that the wave speed will depend not only on the direction of propagation but also on the wave polarization. Particularly, the elastic wave propagation in TI tissue is governed by a set of linear equations known as “Christoffel equations” which relate wave speed, propagation direction, polarization and mechanical properties.

Let us consider a TI tissue that obeys Hooke’s law. In the absence of body forces a disturbance propagating within the medium will obey the following wave equation

$$\rho \frac{\partial^{2}u_{i}}{\partial t^{2}}=C_{ijkl}\frac{\partial^{2}u_{l}}{\partial x_{j}\partial x_{k}}$$ (4)

Where $u_{i}$ ( i = 1, 2, 3) denotes the components of the displacement field and ρ is the material density assumed to be constant ( ∼ 1,100 kg/m^3^). Under plane wave decomposition, a monochromatic plane wave of the form $u_{i}\left(\vec{r},t\right)=u_{o}\exp\left[i\left(\vec{k}.\vec{r}-\omega t\right)\right]$ where the wave amplitude is $u_{o}$ , $\vec{k}$ denotes the wave vector and ω is the frequency is a non-trivial solution of Eq. 4 if the following secular equation is satisfied:

$$\left|C_{ijkl}k_{j}k_{l}-\rho \omega^{2}\delta_{ik}\right|=0$$ (5)

Eq. 5 can be rewritten in terms of the phase velocity v=ω/k , the direction cosines $n_{i}$ ( i = 1, 2, 3) and the Christoffel matrix $\Gamma_{ik}=C_{ijkl}n_{j}n_{l}$ as

$$\left|\Gamma_{ik}-\rho v^{2}\delta_{ik}\right|=0$$ (6)

Eq. 6 corresponds to the eigenvalue equation for matrix Γ and is known as the Christoffel equation. From Eq. 2, we may now write Γ in the particular case of a TI tissue:

$$\Gamma =\left(\begin{array}{ccc} C_{11}n_{1}^{2}+C_{66}n_{2}^{2}+C_{44}n_{3}^{2} & \left(C_{11}+C_{66}\right)n_{2}n_{1} & \left(C_{44}+C_{13}\right)n_{1}n_{3}\\ \left(C_{11}+C_{66}\right)n_{2}n_{1} & C_{66}n_{1}^{2}+C_{11}n_{2}^{2}+C_{44}n_{3}^{2} & \left(C_{44}+C_{13}\right)n_{2}n_{3}\\ \left(C_{44}+C_{13}\right)n_{1}n_{3} & \left(C_{44}+C_{13}\right)n_{2}n_{3} & C_{44}n_{1}^{2}+C_{44}n_{2}^{2}+C_{33}n_{3}^{2} \end{array}\right)$$ (7)

In what follows we will consider some examples of particular interest for shear wave elastography in muscles.

i) $\vec{n}=\left(1,0,0\right)$ : For this case there are three different phase velocities (i.e. three different eigenvalues) given by $v_{1}=\sqrt{C_{11}/\rho }$ , $v_{2}=\sqrt{C_{66}/\rho }$ and $v_{3}=\sqrt{C_{44}/\rho }$ with eigenvectors ${\hat{e}}_{1}=\left(1,0,0\right)$ , ${\hat{e}}_{2}=\left(0,1,0\right)$ and ${\hat{e}}_{3}=\left(0,0,1\right)$ , respectively. The first eigenvector corresponds to a pure longitudinal mode propagating at the speed of ultrasound while the second and third eigenvector correspond to pure shear modes with phase velocities given by the shear moduli $\mu_{T}$ and $\mu_{L}$ , respectively. Consequently, the shear anisotropy can be measured by propagating shear waves in depth and using a source that favors each mode, as it is depicted in Royer et al., 2011.

ii) $\vec{n}=\left(0,\sin\left(\phi \right),\cos\;\left(\phi \right)\;\right)$ : For this example, an evident solution to Eq. 6 is:

$$\rho v^{2}=C_{66}\sin^{2}\left(\phi \right)+C_{44}\cos^{2}\left(\phi \right)$$ (8)

with eigenvector ${\hat{e}}_{1}=\left(1,0,0\right)$ , thus, corresponding to a “pure” shear wave which is the mode often used in most shear wave elastography techniques applied to skeletal muscle. This mode is termed shear horizontal mode (SH) in the sense that the wave polarization vector ${\hat{e}}_{1}$ is perpendicular to the plane formed by the material symmetry axis and wave propagation direction. As it will be seen below there are other ways to generate a SH mode.

It is important to point out that the velocity given by Eq. 8 corresponds to the phase velocity of the wave which is not the magnitude measured by many elastography techniques. Many elastography techniques measure the time of flight of a wave packet through a known distance to compute the wave velocity. This corresponds to the group velocity of the wave packet. In a non-dispersive isotropic medium, the phase and group velocities are identical. However, in a TI tissue the phase and group velocity along the same direction are not necessarily equal. From Eq. 8 it can be shown that the group velocity $v_{g}$ obeys the following equation (Wang et al., 2013):

$$\rho v_{g}^{2}=\frac{C_{44}C_{66}}{C_{44}\sin^{2}\left(\phi \right)+C_{66}\cos^{2}\left(\phi \right)}$$ (9)

iii) $\vec{n}=\left(\sin\left(\theta \right),\;0,\cos\left(\theta \right)\right)$ : This corresponds to the case of shear wave elastography being applied to pennate muscle, particularly, when wave propagation vector forms an angle with the fiber orientation. In this example the eigenvalues are given by:

$${\rho v}_{1,3}^{2}=\frac{1}{2}\left[\Gamma_{11}+\Gamma_{33}\mp \sqrt{{\left(\Gamma_{33}-\Gamma_{11}\right)}^{2}+4\Gamma_{13}^{2}}\right]$$ (10)

(where the minus sign corresponds to the first eigenvalue) and:

$${\rho v}_{2}^{2}=\Gamma_{22}=C_{66}\sin^{2}\left(\theta \right)+C_{44}\cos^{2}\left(\theta \right)$$ (11)

The second eigenvalue corresponds to a pure shear wave, i.e. with eigenvector parallel to ${\hat{x}}_{2}$ while the first and third eigenvalues correspond to a quasi-shear and a quasi-longitudinal wave. In the incompressibility limit the phase velocity for the quasi-shear mode reduces to (Rouze et al., 2013):

$$\rho v_{1}^{2}=\mu_{L}+\left(\frac{E_{L}}{E_{T}}\mu_{T}-\mu_{L}\right)\sin^{2}\left(2\theta \right)=C_{44}+\left(\frac{C_{33}C_{11}-C_{13}^{2}}{4\left(C_{11}-C_{66}\right)}-C_{44}\right)\sin^{2}\left(2\theta \right)$$ (12)

and its polarization is purely transverse. It is important to point out that the incompressibility limit, shear wave propagation in TIT only depends on three mechanical properties: $\mu_{L}$ , $\mu_{T}$ and the anisotropy ratio of the Young’s moduli $\frac{E_{L}}{E_{T}}$ . Thus, all three parameters required to characterize an incompressible TI material can be measured by observing shear wave propagation in the material. Finally, in the recent work of (Rouze et al., 2019) and (Caenen et al., 2020) the following terminology was used to describe both transverse modes: a SH mode (i.e. defined in Eq. 11) and the shear vertical (SV) mode which is defined by a wave polarization vector parallel to this plane (i.e. mode corresponding to the first eigenvalue in the incompressibility limit).

In the next parts, we will describe the most representative work allowing the quantification of biological tissues anisotropy using ultrasound elastography techniques, from 1D to 3D elastography in transverse isotropic medium.

## 3 Anisotropy quantification by elastography methods

### 3.1 1D elastography

**FIGURE 1:**
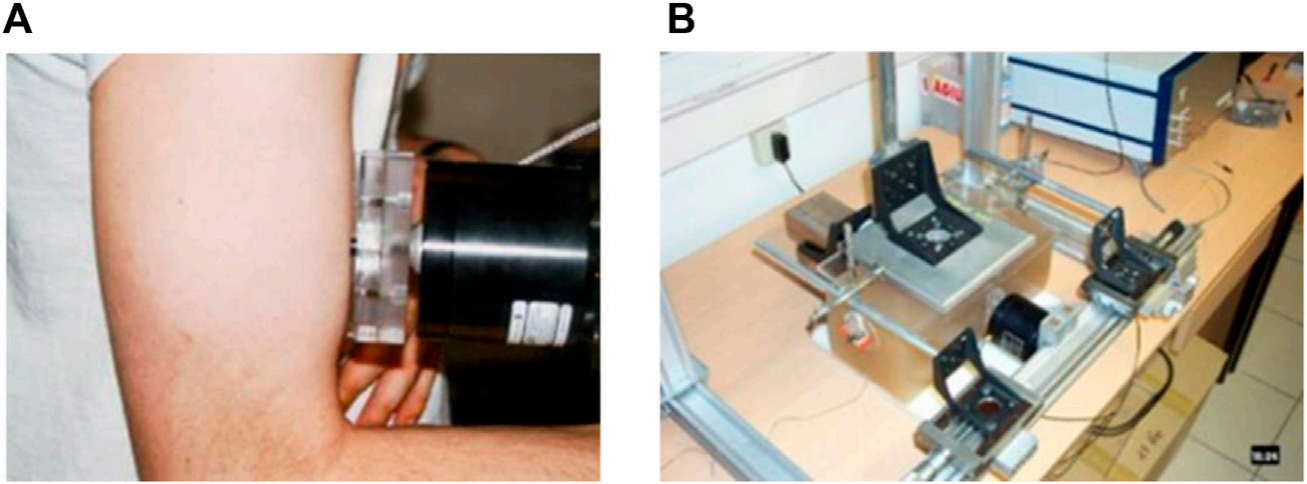
**(A)** 1D shear elasticity probe applied *in vivo* on the *biceps brachii* to quantify stiffness anisotropy by rotating the rod fixed on the mini-shaker. **(B)** Apparent anisotropy quantified with two 1D shear elasticity probe in an acoustoelasticity experiment on agar-gelatin phantom.

1D elastography is, to our knowledge, the first dynamic ultrasound elastography technique that was developed to quantify elastic anisotropy through the use of the shear elasticity probe (Gennisson et al., 2003). The idea was to change the polarization of shear waves to measure the shear modulus along and perpendicular to fibers. Gennisson et al. (2003) proposed to use a rod fixed on a vibrator to increase the energy of shear waves perpendicularly to its axis of percussion. Then, by placing the probe parallel or perpendicular to the muscle fibers, the shear waves speed could be retrieved by following the theoretical part described in Section 2 (Eq. 8). This allow the quantification of muscle stiffness *ex vivo* (Gennisson et al., 2003) and *in vivo* (Gennisson et al., 2005). This device is presented in Figure 1A on an *in vivo* experiment on the *biceps brachii*. Figure 1B displays the device allowing the estimation of the elastic non-linear shear parameters in an acoustoelasticity experiment by quantifying the apparent anisotropy induced by a uniaxial stress applied on the medium (Catheline et al., 2003).

To improve this 1D approach, Chatelin et al. (2014a) proposed a new remote shear wave elasticity probe for anisotropy investigation based on radiation force of a focused ultrasound beam. The previous mini-shaker that generates shear waves on the shear elasticity probe was replaced by a focused transducer that will create remotely and locally within the muscle a shear wave source (Sarvazyan et al., 1998). Closely to this focused transducer, 8 single transducers were smartly placed to track the shear wave propagation parallel and perpendicular to the muscle fibers (Tanter et al., 2016). Taking advantage of this first prototype, Pedreira et al. (2021) recently developed a new compact probe to investigate stiffness anisotropy of the myocardium. The transducer is composed of five concentric annular elements and three lateral elements. The annular elements allow the generation of an acoustic radiation force at a chosen depth and display the M-mode images of the region of interest for a better positioning of the probe. Lateral elements are angularly spaced at 120° to estimate the local displacement in the medium around the pushing zone. Shear velocities along and across the fibers are finally assessed thanks to the ellipse fitting of the propagating shear wave using Eq. 9.

### 3.2 2D elastography

Complementary to 1D elastography, 2D elastography devices were developed using different approaches. As for 1D elastography, multiple shear wave sources were proposed leading to different 2D elastography methods, most of which are presented here:

#### 3.2.1 Transient elastography

In 2004, Catheline proposed to adapt 1D transient elastography to anisotropic measurements in 2D. A plate was fixed on a mini-shaker to generate 2D plane shear waves in a muscle. Shear waves were then caught in 2D by an ultrafast ultrasound probe composed of 128 channels. By placing the shaking plate along or perpendicular to muscle fibers, they deduced both shear moduli along two axes of an *ex vivo* muscle (Catheline et al., 2004).

#### 3.2.2 Supersonic shear imaging

**FIGURE 2:**
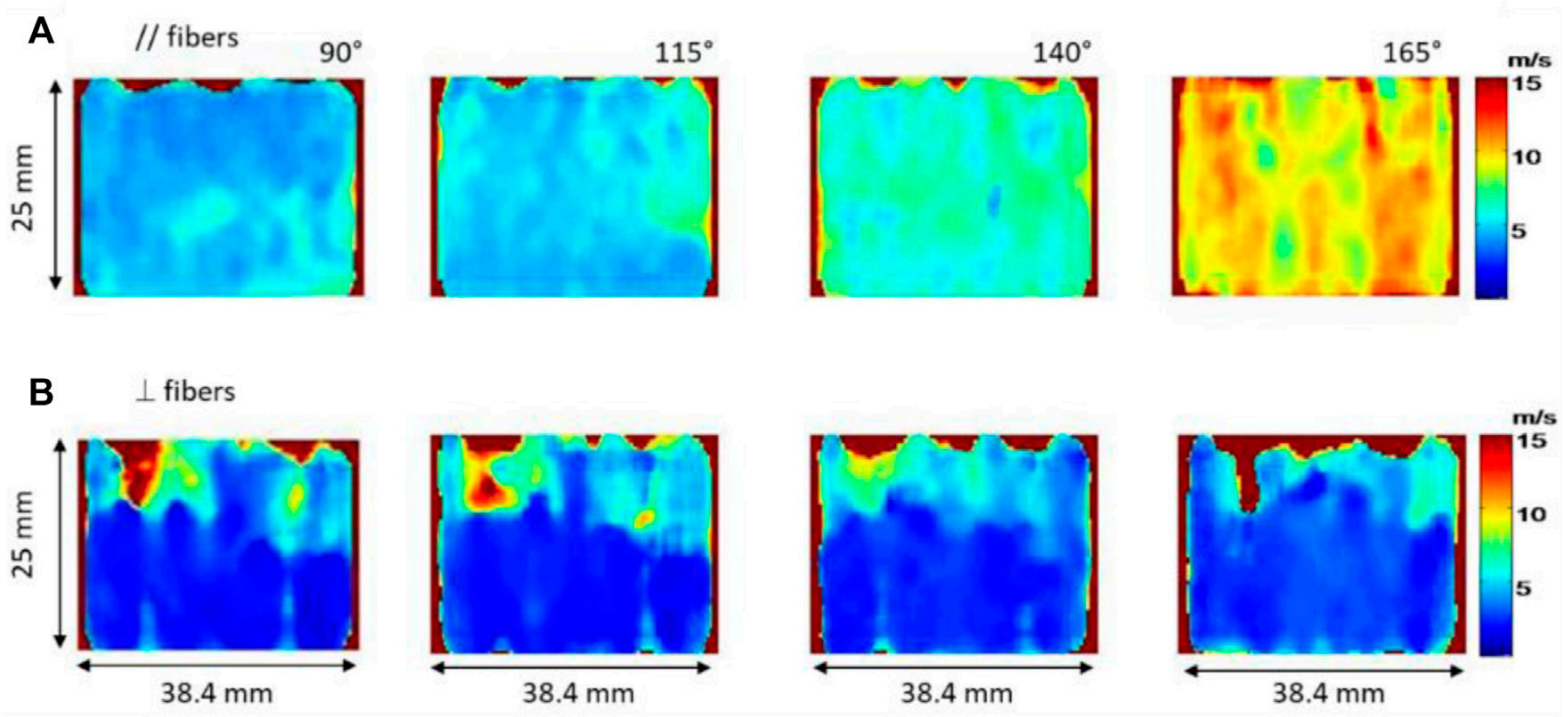
Shear wave velocity maps of the biceps brachii for passive extension of the elbow with different angle (from 90° to 165° with 25° step) along the fibers **(A)** and perpendicularly to the fibers **(B)**.

The development of the Supersonic Shear Imaging (SSI) technique (Bercoff et al., 2004) is a major breakthrough in the field of 2D elastography. Briefly, this technique consists in generating shear waves by using the acoustic radiation force and catching their propagation by using ultrafast imaging that allows an imaging frequency up to 20 kHz. Gennisson et al. (2010) were, to our knowledge, the first to quantify muscle anisotropy in 2D. Two muscles were studied: human *biceps brachii* and *brachialis*, which are fusiform muscles parallel to each other. Stiffness of both muscles were measured during isometric contractions and passive extensions (Figure 2) by using two probe positions: parallel and perpendicular to the fibers. In this specific case the shear wave group velocity was investigated as described in the theoretical part Section 2. Shear waves group velocity was measured at different levels of isometric contraction. As a result, sets of shear wave group velocity maps of two adjacent muscles oriented differently with different degrees of stress (isometric contraction and passive extension) were produced in both orientations of the shear wave propagation. The relationship between elasticity and anisotropy was then studied. The authors found that shear wave group velocity progressively decreased as the probe was more and more perpendicular to muscle fibers. More so, experimentally it was observed that this decrease was even more pronounced in the contracted state than in the relaxed state. It was thus deduced that the evaluation of muscle anisotropy would be more evident in the contracted state than the relaxed one. Through these measurements, the muscle anisotropic mechanical properties can be assessed with a complete set of quantitative data.

**FIGURE 3:**
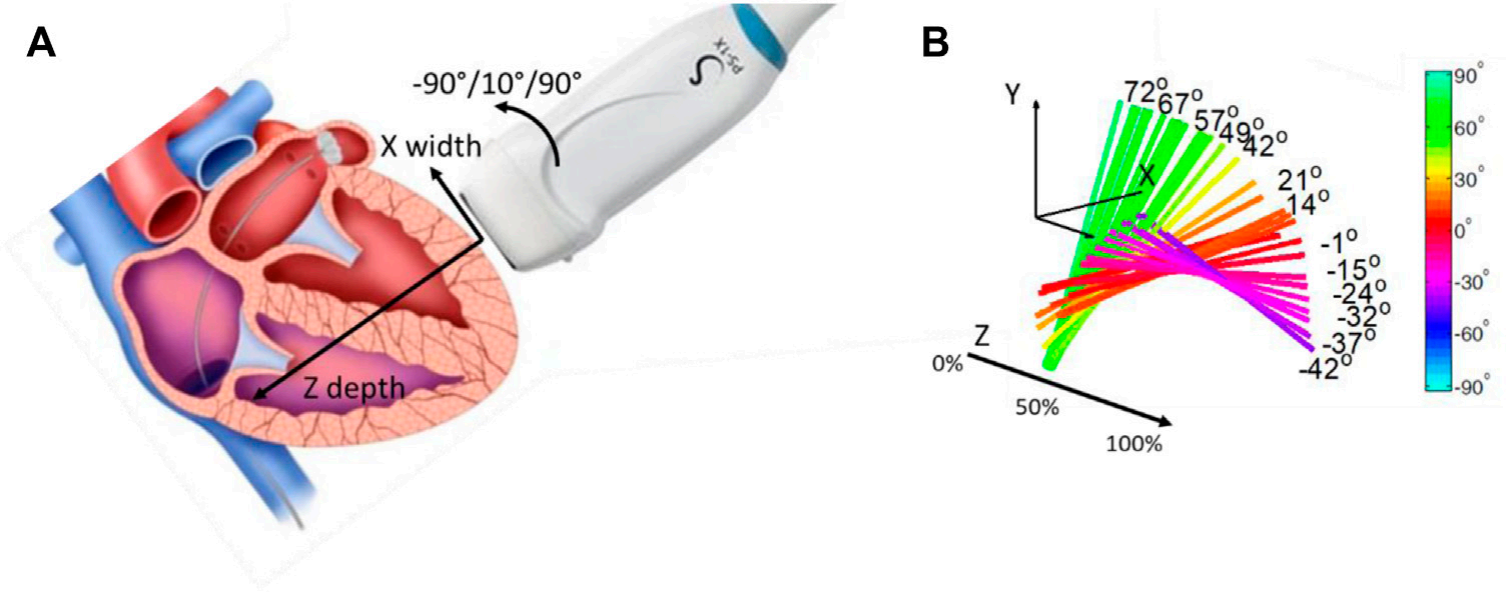
**(A)** Probe positioning regarding the myocardium wall. The probe can rotate following the Z axis, depth of the myocardium wall. **(B)** Fiber orientation for each depth of the myocardium wall, in percentage of the wall thickness.

In 2012, to validate the use of the SSI method as a tool to quantify anisotropy, Lee et al. (2012a); Lee et al. (2012b) investigated myocardium fiber orientation. Myocardium is structured in distinct layers with varying orientations across the heart wall (Streeter and Bassett 1966; Streeter et al., 1969). In this work, the ultrasonic probe was placed at the surface of an *ex vivo* myocardium and rotated around a single axis (Figure 3A). Shear waves were generated by using acoustic radiation force and shear wave velocities were measured for different depths within the myocardium for each angle of rotation (Figure 3B). By looking at the maximum velocity as a function of the angle for each depth, the muscle fiber orientation was retrieved. Results were compared to diffusion tensor imaging by using magnetic resonance imaging, a technique allowing the tracking of fibers orientation in space. A good agreement between the two techniques showed that this ultrasonic approach, so-called elastic tensor imaging, can be proposed for the assessment of the fiber structure in muscle.

Following the same method of a pushing beam followed by an ultrafast acquisition, Urban et al. (2015) used in 2016 a transesophageal ultrasonic probe to generate in an *ex vivo* myocardium shear waves. By using an ultrafast device, they quantified shear wave velocity at different frequencies (50, 100 and 200 Hz) for different angle of the probe regarding the muscle fibers (from 0° to 170° with 9° increment). They found that low frequencies yield low sensitivity to the fiber’s orientation and the true wave speeds in the layers was underestimated. By using excitations at higher frequencies, a better estimation of the fiber orientation and more accurate shear wave speeds measurement was possible. This work provided the understanding that the anisotropy factor is clearly better determined at high shear wave frequencies.

#### 3.2.3 Shear wave ultrasound vibrometry

In 2009, Chen et al. proposed to investigate anisotropy in an *ex vivo* muscle by using the Shear wave Dispersion Ultrasound Vibrometry (SDUV) technique. The basic principle of SDUV is to generate a harmonic shear wave by an ultrasound beam (*i.e.,* push), and its propagation is monitored by a separate ultrasound beam (*i.e.,* detection beam). The shear wave speed is calculated from its phase measured at 2 different locations separated by a known distance along its traveling path (Chen et al., 2009). The results of this study were compared with finite element simulations. This technique was also used to measure the viscoelastic properties of *ex vivo* bovine and porcine striated muscles (Urban et al., 2009; Urban and Greenleaf, 2009). In these studies, the measurements were performed along and across the muscle fibers. As an example, in the bovine muscle tissue, the shear modulus values retrieved were *µ*_*//*_ = 29 kPa and *µ*_*⊥*_ = 12 kPa, measured along and across the muscle fibers, respectively. In the porcine muscle tissue, the values were *µ*_*//*_ *=* 12.7 kPa and *µ*_*⊥*_ = 5.3 kPa, measured along and across the muscle fibers, respectively. The studies showed an anisotropic ratio of about 2–3 when performing the ratio of shear waves speed along and across muscle fibers.

#### 3.2.4 Acoustic radiation force impulse—shear wave speed

In parallel to the development of supersonic shear imagine technique, Nightingale et al. (2003) proposed in 2003 to use acoustic radiation force impulse as a source for shear wave generation. The principal difference with supersonic imagine was the way to image the propagation of shear waves by using parallel beamforming over a small number of tracking lines close to the pushing beam. In 2013, Rouze et al. (2013) developed a finite element simulation of shear wave propagation in an incompressible transversely isotropic medium opening the way to use the ARFI-SWS technique in studies. Recently, Rouze et al. (2020) investigated a new modeling technique using the Green’s tensor method to better characterize this anisotropy (Rouze et al., 2020). The Green’s tensor is expressed with an analytic expression of SH mode and an integral expression of SV mode which allow to reduce the number of numerical integrals by a factor up to 10^9^, significantly lessening computational complexity. The SV and SH propagation mode were defined by the relative orientation of waves polarization with respect to the plan formed by waves propagation and material symmetry axis. This method is able to provide the three parameters that characterize an incompressible and TI material: the longitudinal *µ*_*//*_ and transverse *µ*_*⊥*_ shear modulus, and the tensile anisotropy *χ*_*E*_ (the ratio between the Young’s modulus parallel and the Young’s modulus perpendicular to the fibers) following an ARFI-SWS. Taking advantage of this modeling method, ultrasonic rotational 3D elasticity imaging was developed to fully characterize muscle mechanical properties (Knight et al., 2021). To estimate the three mentioned parameters, both SH and SV are measured by rotating the probe in 3D. Regarding the modes, only *χ*_*E*_ has an impact on the SV propagation. *In vivo* human experiments were conducted on the *vastus lateralis*, a pennate muscle. That allows to experimentally identify group SH and SV velocity using the Radon sum algorithm (Rouze et al., 2010). By fitting these experimental values to Eqs. 11, 12, all three transverse isotropic material parameters were computed.

### 3.3 3D elastography

To palliate the problem of being restrained to a 1D linear transducer that move or rotate to get a full 2D elastography volume, Wang et al. (2013) combined a spherical transducer, used to generate a shear wave by ARFI, with a 2-D matrix array to image shear wave propagation. By imaging the propagation of the shear wave plane by plane in a sequential way, they were able to synthetically reconstruct the shear wave propagation in the entire volume. One advantage of this technique is that the pushing beam is maintained at the same location while the tracking beams are shifted in different directions, which effectively allows for the quantification of the elastic anisotropy. However, because acquisitions must be repeated sequentially to acquire the full volume, the technique yields a low volumetric frame rate and as such is limited in presence of motion due, e.g., to breathing or pulsatility. In Provost et al. (2014) developed further away the concept of ultrafast ultrasound imaging to 2D matrix array by designing a 3D ultrafast ultrasound device driving 1,024 channels. With intuitive beamforming reconstruction applied to the small aperture of 2D matrix array, Provost et al. (2014) proposed new ways to image the full volume of large organs in real time such as the heart. Later, Gennisson et al. (2015) took advantage of this new ultrafast device to proposed the first full 3D shear wave elastography probe with an ultrafast ultrasound device. Therefore, shear wave propagation can be induced and imaged in three-dimensions, and tissue stiffness can be estimated in a full volume. Experiments were performed in an isotropic breast phantom dedicated to elastography and *in vivo* measurements were also performed on a healthy volunteer in the upper right quadrant of the breast. Correia et al. (2018) then showed that anisotropy was quantifiable with this approach in three-dimensions, at high volume rate and in a few minutes of post-processing. The technique was evaluated numerically in simulated transverse isotropic model with different degrees of stiffness and anisotropy. They showed good results in weakly transversely isotropic media (PVA phantoms, Chatelin et al., 2014b) and quantification of low fractional anisotropy (< 0.34) was validated with numeric simulation.

## 4 Discussion

Shear wave ultrasound elastography is an important research field and has been growingly used in a variety of clinical settings over the last 15 years. It has provided a set of ultrasound methods allowing the noninvasive assessment of tissue stiffness *in vivo.* Several studies have shown that tissue stiffness is of great value for medical diagnosis. These advantages should certainly lead to new applications of shear wave elasticity imaging, not only for diagnosis but also for the follow-up of tissue mechanical properties in a wide range of population, whether it is athletes or patients affected with neuromuscular dystrophies to name a few. The real-time capability of some of these shear wave techniques also allowed the development of 3D elastography imaging that should facilitate the clinical use for detection, therapy planning and monitoring in the routine clinical practice.

In all these approaches, tissues are assumed to be isotropic and the variation of the shear modulus in biological tissues offers a contrast that is potentially more interesting than conventional ultrasound. This was possible because researchers have decrypted the underlying physics of each shear wave elastography method to understand their advantages and physical limitations. In this paper, we have highlighted the available shear wave elastography methods which brought effective tools that was enhanced to define new biomarker such as anisotropy. We depicted the methods for muscular elastic anisotropy characterization from the simplest in 1D to the more complex in 3D.

All methods have their advantages, such low cost in 1D elastography (Pedreira et al., 2021) or full characterization in 3D elastography (Correia et al., 2018), and drawbacks, such as the rotation step in 2D (Gennisson et al., 2010), but all allow the determination of tissue anisotropy. Globally, only the simplest case of anisotropy, TI medium were examined. This anisotropy is easily investigable in fusiform muscle such as *biceps brachii*. Otherwise, muscles have diverse arrangements which are much more complicated than parallel structures (fusiform) with notably higher anisotropic degree, namely, circular muscles (*orbicularis oris* muscle), convergent muscles (*pectoralis major*) or bipennate muscle (*rectus femoris*), etc. Further research on different muscle models is essential to achieve a powerful method of 3D elastography that considers all the possible muscle anisotropies. Finally, all available elastography methods are unable to provide information on the orientation of the fibers in the elevation dimension (shear waves are always polarized in this axis of the ultrasound). This issue must be carefully studied to improve the 3D elastic maps.

For this biomarker, anisotropy, to be clinically relevant and usable, its quantification needs to be facilitated. For example, 2D elastography can be used to quantify tissue anisotropy in 3D by performing multiple recordings with various probe angle. In the case of fusiform muscle, if the probe is placed parallel to fibers, with a rotation around the axis defined by the ultrasound beam it is possible to determine the fiber orientation with only shear wave velocity measurements. However, in pennate muscle shear wave velocity measurements must be supplemented with Bmode acquisition in order to retrieve fiber orientation. Here 3D rotation is definitively needed as demonstrated by Knight et al., 2021. However, probe rotation is a major drawback and is not applicable to a muscle with constantly changing contraction levels. 3D elastography appears to bridge the gap between these limitations and what is expected from clinicians but still requires expensive device that are rarely if ever available in a clinical setting. So the last solution would be to use a 2D device that is fast enough to asses locally anisotropy but limits the measurement to one point, which can be a problem for full muscle characterization in specific pathologies such as myopathies. Considering all these advantages and drawbacks of these techniques, the ultimate goal would be to find a way to quantitatively estimate muscle anisotropy in real time in a 2D imaging plane without moving the probe.

## 5 Conclusion

Over the last 20 years, ultrasound elastography have been massively studied and deployed in various clinical and scientific settings. Beside stiffness, ultrasound elastography allows the assessment of tissue anisotropy, whether by performing multiple acquisitions using 1D or 2D elastography, or a single acquisition using 3D elastography. Tissue anisotropy may be a very relevant biomarker of tissue mechanical properties and could improve our understanding of muscle adaptations during aging, training, or diseases. Further developments should mainly focus on the implementation of readily available ultrasound elastography sequences that would allow clinicians and specialist to monitor tissue anisotropy on a daily basis.
